# Tool for Eliminating Dog-Mediated Human Rabies through Mass Dog Vaccination Campaigns

**DOI:** 10.3201/eid2312.171148

**Published:** 2017-12

**Authors:** Eduardo A. Undurraga, Jesse D. Blanton, S.M. Thumbi, Athman Mwatondo, Mathew Muturi, Ryan M. Wallace

**Affiliations:** Centers for Disease Control and Prevention, Atlanta, Georgia, USA (E.A. Undurraga, J.D. Blanton, R.M. Wallace);; Kenya Medical Research Institute, Nairobi, Kenya (S.M. Thumbi);; Washington State University, Pullman, Washington, USA (S.M. Thumbi);; Kenya Ministry of Health, Nairobi (A. Mwatondo, M. Muturi)

**Keywords:** rabies, economics, vaccination, prevention, elimination, viruses, Kenya, zoonoses, dogs

## Abstract

The World Health Organization and collaborating agencies have set the goal of eliminating dog-mediated human rabies by 2030. Building on experience with rabies endemic countries, we constructed a user-friendly tool to help public health officials plan the resources needed to achieve this goal through mass vaccination of dogs.

Globally, rabies kills ≈60,000 persons annually; most (≈99%) cases are transmitted by domestic dogs (*1*–*3*). Controlling dog rabies through periodic mass vaccination campaigns substantially reduces human exposures (*4*). The elimination of dog rabies in most of the Western Hemisphere and countries in Asia has demonstrated the effectiveness and sustainability of vaccinating dogs (*5*,*6*) by combining massive dog rabies vaccination with coordinated efforts of the medical and veterinary sectors (One Health approach), including education about responsible pet ownership, rabies awareness campaigns, and access to postexposure prophylaxis (*5*,*6*).

The World Health Organization recommends that at least 70% of the dog population be vaccinated to control and potentially eliminate dog rabies (*3*). In 2016, WHO and partner organizations set the goal of eliminating dog-mediated human rabies by 2030 (*7*). This goal could be achieved by massive, costly administration of preexposure and postexposure prophylaxis, mass vaccination of dogs, or both.

Countries to which rabies is endemic in dogs are at different stages in their rabies control efforts (*5*,*8*). Countries at early stages face barriers related to a limited understanding of the local epidemiology, logistic and operational challenges, competing priorities from other diseases, and lack of planning tools to reasonably project the resources needed. During 2016, we estimated the resources potentially required to eliminate dog rabies globally by 2030 (*7*). We combined multiple data sources to estimate 4 key factors that affect this goal: country development, cost of dog vaccination programs, potential demand for dog rabies vaccine, and estimated number of vaccinators. We aimed to realistically assess the global situation by highlighting the main challenges that might hamper elimination efforts. However, although these global estimates can inform an important discussion about global and regional strategic planning and resource mobilization, they are not necessarily useful to inform country-level decision-making. We addressed this limitation by providing a user-friendly tool that requires only limited country-specific data to help countries plan toward the goal of eliminating dog rabies through mass dog vaccination.

We designed the tool to plan for dog rabies elimination by 2030 (a 13-year framework). We assumed 13 years would be enough time for even the least developed rabies control programs to achieve elimination, provided the country was fully committed. The country’s starting point within this time frame would depend on its current dog vaccination rate, and the given country would demonstrate incremental improvements in preparation for the vaccination campaign (e.g., training of workforce involved, dog population surveys) or in the proportion of the dog population vaccinated. The tool requires input of demographic data (human population, percentage urban, human-to-dog ratio); current dog vaccination coverage; logistic data for the campaigns (available vaccinators, dog vaccination rates, campaign duration); and an estimated cost per vaccinated dog. We constructed a worksheet to help users estimate the cost per vaccinated dog based on a pilot campaign. The tool (including assumptions, instructions, and data requirements) is available for public use (Technical Appendix) and already has been used in Haiti and Guatemala as part of a rabies elimination workshop held at the Centers for Disease Control and Prevention (Atlanta, GA, USA). Users can account for uncertainty in point estimates by varying model parameters.

To show the utility of the planning tool, we input known information from 2 recent mass dog vaccination campaigns in western Kenya to project expected long-term costs and a timeline for rabies elimination. With a conservative estimate of 523 (95% CI 138–1,100) annual human rabies-associated deaths and substantial medical costs (*1*), Kenya is actively trying to eliminate human rabies by 2030 (*9*). We used the following values: 46,050,302 persons, of whom 26% live in urban areas; 7.4 and 21.2 humans per dog in rural and urban areas, respectively; 5% of the dog population vaccinated; 4,300 available vaccinators; 100 dogs vaccinated daily per vaccinator; a 21-day campaign in each region; an estimated cost per dog vaccinated of US $1.81 (range US $1.48–$2.12); and 0% discount rate (Figure). Conditional on the availability of resources, the results suggest that Kenya can eliminate dog rabies during the next 13 years, in line with the 2030 global goal; vaccination campaigns would cost ≈US $62.0 million (range US $50.6 million–$72.8 million), equivalent to ≈$56.0 million additional aggregate spending over 13 years (not discounted). If the average vaccination rate was 100 dogs per vaccinator per day, Kenya would have enough capacity to conduct 21-day campaigns and reach the 70% dog vaccination goal.

**Figure:**
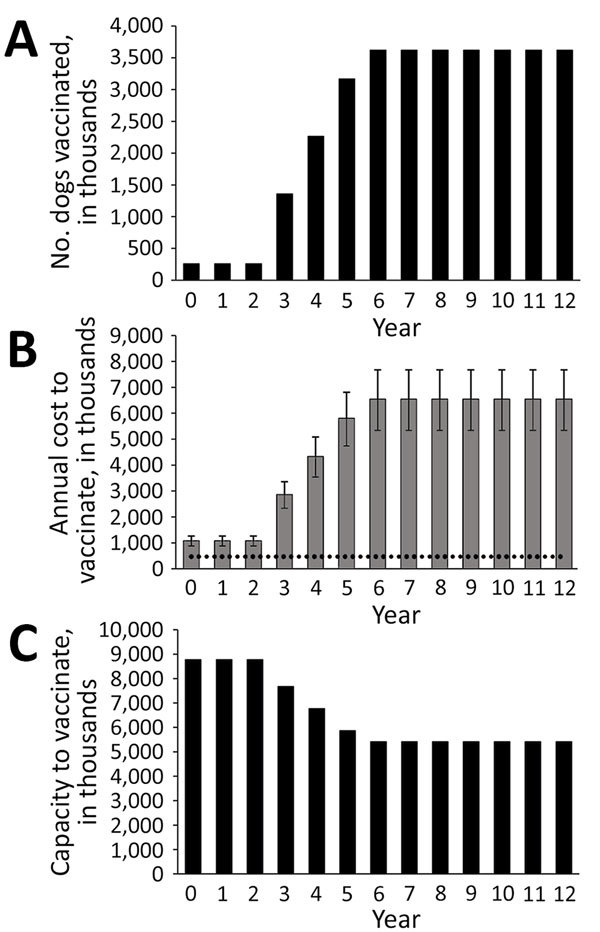
Illustrative results from the planning aid tool for controlling dog rabies through dog vaccination using input data, Kenya, 2016. A) Number of annual dog vaccinations required in accordance with World Health Organization recommendations. The tool assumes a threshold of 70% of dog vaccination during 7 years as a conservative estimate to eliminate dog-mediated rabies. The actual proportion of the dog population that needs to be vaccinated depends on local conditions of rabies transmission (*10*). Based on field data, Kenya estimates that 3 consecutive years of 70% coverage of dog vaccination would end dog–dog rabies transmission. The estimate by Hampson et al. (*1*) estimate of 523 annual deaths was based on active surveillance in eastern Kenya; current passive surveillance reports higher bite rates, so the estimate probably represents a lower bound of the number of deaths that could be avoided through mass dog vaccination. B) Total annual cost (US $) of dog vaccination. Error bars indicate 95% CIs. Horizontal dotted line indicates current spending for dog vaccination. C) Net dog vaccination capacity (i.e., total number of dogs per year minus the number of dogs Kenya needs to vaccinate to achieve the dog vaccination coverage goal). If the number is positive, the country has enough capacity to vaccinate; if negative, the country needs more vaccinators, increased vaccination efficiency, or more campaign days. The estimated annual costs are based on the cost per dog vaccinated; excess vaccinator capacity is not included in the aggregate costs.

The tool enables users to estimate the resources required to eliminate rabies using country-specific input values. We hope this tool will help stimulate and inform a necessary discussion on strategic planning, resource mobilization, and continuous execution of rabies elimination.

Technical Appendix Planning tool for controlling dog-mediated human rabies deaths by dog vaccination.
